# Phenotypic plasticity of coralline algae in a High CO_2_ world

**DOI:** 10.1002/ece3.723

**Published:** 2013-08-28

**Authors:** Federica Ragazzola, Laura C Foster, Armin U Form, Janina Büscher, Thor H Hansteen, Jan Fietzke

**Affiliations:** 1 GEOMAR, Helmholtz Centre for Ocean ResearchWischhofstraße 1-3, 24148, Kiel, Germany; 2 Department of Earth Sciences, University of BristolWills Memorial Building, Queen's Road, BS8 1RJ, U.K

**Keywords:** Climate change, coralline algae, long-term experiments, ocean acidification

## Abstract

It is important to understand how marine calcifying organisms may acclimatize to ocean acidification to assess their survival over the coming century. We cultured the cold water coralline algae, *Lithothamnion glaciale,* under elevated *p*CO_2_ (408, 566, 770, and 1024 μatm) for 10 months. The results show that the cell (inter and intra) wall thickness is maintained, but there is a reduction in growth rate (linear extension) at all elevated *p*CO_2_. Furthermore a decrease in Mg content at the two highest CO_2_ treatments was observed. Comparison between our data and that at 3 months from the same long-term experiment shows that the acclimation differs over time since at 3 months, the samples cultured under high *p*CO_2_ showed a reduction in the cell (inter and intra) wall thickness but a maintained growth rate. This suggests a reallocation of the energy budget between 3 and 10 months and highlights the high degree plasticity that is present. This might provide a selective advantage in future high CO_2_ world.

## Introduction

The rise of anthropogenic emissions of CO_2_ is altering the ocean chemistry (ocean acidification) with a decrease in average seawater pH by 0.1 units since the last century and with a projected drop of another 0.3–0.4 units by 2100 (Solomon et al. 2007). This translates to a lowering of the calcium carbonate saturation state of the seawater (Ω) which may compromise the ability of many marine calcifying organisms to form their calcium carbonate shells or skeletons (Orr et al. 2005; Doney et al. 2009).

Within the shallow water ecosystems dominated by macroalgae, those consisting of coralline algae are not only the most common (Foster 2001) but also the most widespread framework builders north and south of the low latitude coral belt (Freiwald and Henrich 1994). They increase benthic diversity by providing a hard substrate with a complex architecture (Guillou et al. 2002; Wilson et al. 2004) and are a significant component of the global carbon system (Mackenzie et al. 2004; Martin et al., 2007) accounting for approximately 25% of CaCO_3_ accumulation within coastal regions (Martin et al., 2007). In tropical regions, coralline algae have been shown to be a major contributor in the formation and stabilization of coral reefs and enhancing the coral larvae settlement (Chisholm 2000). Due to their crucial role in shallow water ecosystem and their worldwide distribution, understanding the impact of ocean acidification on calcifying algae is fundamental. Furthermore, their high-Mg calcite skeleton is the most soluble polymorph of CaCO_3_ (50% more soluble than calcite and 20% more soluble then aragonite) and thus they are likely to be particularly sensitive to a reduction in Ω (Busenberg and Plummer 1989; Feely et al. 2004; Andersson et al. 2008). Although most of the studies have highlighted the negative impact of ocean acidification on calcification, ultrastructure, growth, settlement, and abundance (Anthony et al. 2008; Kuffner et al. 2008; Martin and Gattuso 2009; Ragazzola et al. 2012), a few have reported either no change or a increase in coralline algae growth (Martin et al. 2013; Burdett et al. 2012; Ries 2011). CO_2_ has been shown to enhance photosynthesis in different macroalgae (Reiskind et al. 1988; Semesi et al. 2009) and thus making the long-term trade-off between these physiological responses is difficult to predict. At an ecosystem level, ocean acidification also is predicted to increase the susceptibility of algae to grazing and erosion (Andersson and Gledhill 2012; Andersson and Mackenzie 2012; Johnson et al. 2012; Ragazzola et al. 2012), and can potentially lead to a shift toward an ecosystem dominated by noncalcifying species (Hall-Spencer et al. 2008; Kuffner et al. 2008). Species with wide geographic ranges, such as coralline algae, are in general very plastic and able to acclimatize to a variety of habitats through morphological and functional responses (Brody 2004). Phenotypic plasticity of coralline algae has been reported in transplant experiments with morphological changes in response to mechanical forces such as waves and currents (Tyrrel and Johansen 1995; Enriquez et al. 2009). There is a close relationship between the external environment and physiological processes, whether they are essential nutrients and light (Niklas 2009) or changes in seawater chemistry.

Recent short-term experiments have shown a decrease in Mg in the skeleton of several calcifying organisms, including the coralline algae *Neogoniolithon sp*., due to changes in carbonate chemistry (Ries 2011). This change in mineralogy shows a potential plastic response of the organisms but the extent to which such changes influence their ability to sustain calcification is still unclear. In addition, the Mg content is influenced by temperature, and has been used as a paleotemperature proxy (Halfar et al. 2000, 2009; Kamenos et al. 2008; Hetzinger et al. 2009). This proxy is based on the fact that Mg substitution is favored at higher temperatures (Lea 2003) but changes in the saturation state could influence any temperature reconstruction (Ries 2011).

To study the potential of coralline algae to acclimatize to ocean acidification, we cultured *Lithothamnion glaciale*, one of the main maerl-forming species in the northern latitudes, under different elevated CO_2_ levels for 10 months. One of the key issues in ocean acidification is the applicability and length of experiment (short-term vs. long-term experiments). Short-term experiments, including “shock-experiments” (where the organisms are immediately immersed in high CO_2_) may provide physiological information on the reaction to sudden stress, but doesn't indicate the acclimation potential of the organism over longer timescales in a high CO_2_ environment. The definition of a long-term experiment may vary between organisms and interspecies, and it is often difficult to predict how long an experiment must be to see the final acclimatization. A number of experiments in cold water corals and coralline algae have highlighted a differing response between “short-term” and “long-term” experiments (Martin et al., 2007; Kuffner et al. 2008; Form and Riebesell 2012; Martin et al. 2013). This 10-month experiment is the culmination of a long-term experiment, with samples also taken after 3 months (Ragazzola et al. 2012). By comparing the response from these two time intervals, we can determine if different phases of acclimatization occur. We examine the growth rates, cell wall thickness, and volume of calcite to determine the calcification response and modification of the skeleton structure. In addition, we examine the Mg content in *L. glaciale* after 10 months in order to investigate the impact of ocean acidification in the mineralogy of its skeleton and to assess the mineralogical plasticity.

## Material and Methods

As the 10-month experiment reported in the present study is a continuation of the previously published 3-month experiment the material collection, experimental setup, seawater chemistry, and nutrient monitoring are explained in detail in Ragazzola et al. 2012. A summary is provided below.

### Material collection

The samples were collected in Kattegat (57°0.84'N, 11°35.10'E and 57°0.38'N, 11°34.88'E) in June 2010 at a depth of 20 m by RC Littorina using a mini dredge. Only healthy specimens (no sign of damage or bleaching) were selected for culturing. The thalli were stained using 0.5 g/L Alizarin Red S (Fluka, Sigma-Aldrich, Steinheim, Germany) for 24 h at 8°C in 12:12 h light–dark cycle (Blake and Maggs 2003) in an aerated 15 liters tank. The algae were removed and rinsed to remove excess staining before being placed in the aquaria. Postexperimental analyses revealed that the stain was only clearly identifiable in ∼30% of the samples. The low percentage of stained thalli was previously recorded for the same species (Blake and Maggs 2003).

### Experimental setup

This experiment represents a continuation of the experiment that was described by Ragazzola et al. (2012). Some of the algae were removed from the aquaria after 3 months, while in this study the algae were collected after 10 months. Thus, both experiments ran simultaneously and under the same conditions.

The specimens were randomly assigned in sixteen 5 L glass aquaria filled with natural seawater (salinity 32) and bubbled with four different CO_2_ concentrations (410, 560, 640, and 1120 μatm) for 10 months using a CO_2_ mixing-facility (KICO2 – Kiel CO_2_ manipulation experimental facility, Linde Gas & HTK Hamburg, Germany). At the beginning, all aquaria with the algae were supplied with ambient air (*p*CO_2_ of ∼422 μatm) for 1 month (and then were stained). After taking initial water samples for total alkalinity (TA), the CO_2_ concentration were slowly increased at the same rate for all treatments (over a maximum of 1 month) until the desired concentrations were reached. The experimental conditions were set at 7 ± 0.5°C with 20 μmol photons m^−2^ sec^−1^ (as found at 20 m at Kattegat during the summer season) in a 12-h light/dark cycle. The water was exchanged approximately fortnightly (as required) so that the nutrient levels and alkalinity were kept constant. All aquaria belonging to the same treatment had the water changed at the same time from a water reservoir, which was adjusted to the treatment *p*CO_2_ concentration by bubbling in CO_2_ over 24 h.

### Monitoring of the carbonate system and nutrients

The same procedure for monitoring was used as described by Ragazzola et al. (2012). Salinity, temperature, pH were measured once a week using a glass combination electrode (SenTix80, WTW, Weilheim Germany) with an accuracy of ±0.005 and a WTW conductometer (LF340) with a TetraCon 325 measuring cell (WTW, Weilheim, Germany). Water samples for the TA were collected every 2 weeks. The carbonate system was calculated from pH and TA measurements using the thermodynamic constants of Mehrbach et al. (1973) as refitted by Dickson and Millero (1987) on the free scale and the carbonate chemistry calculation program CO2SYS (Lewis and Wallace^1998^). These parameters, together with the *p*CO_2_ for the 3-month experiment are shown in Table 1. Water samples for nutrients (nitrate, nitrite, phosphate, and ammonium) were measured photometrically (U-2000; Hitachi, Tokyo, Japan) according to Hansen and Koroleff (1999) with precision levels of ±0.5 μmol/kg, ±0.02 μmol/L, and ±0.05 μmol/L, respectively. Ammonium was measured fluorometrically (SFM 25, Kontron Instruments, Neufahrn, Germany) according to Holmes et al. (1999) with a precision of ±0.08 μmol/L.

**Table 1:** Carbonate system

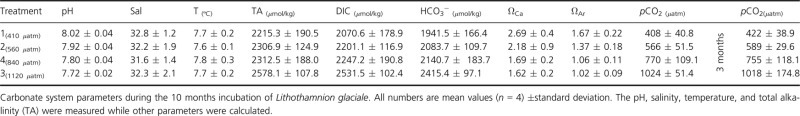

### Growth rate

For each treatment, four samples were sectioned along the longitudinal axis. The linear growth rate was then calculated using ImageJ analysis software (Rasband 2008) by measuring the distance from the Alizarin Red-S stain to the proximal growth edge.

### Scanning electron microscopy

**Figure 1:**
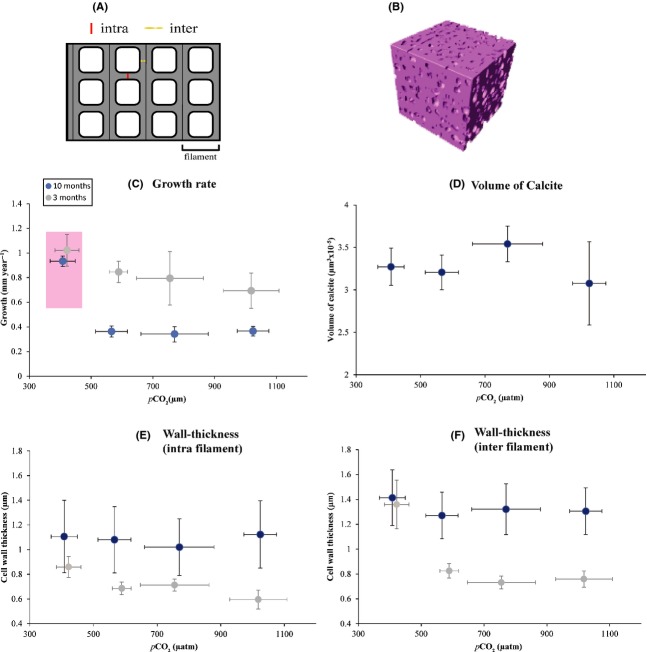
Parameters of growth and calcification of *Lithothamnion glaciale* under four different *p*CO_2_ levels (A) scale model of *L. glaciale* structure showing position of intra- and interfilament walls, (B) example of extracted synchrotron X-ray tomographic microscopy cube from Avizo™ of the control used to determine volume of calcite, (C) linear growth rate, (D) volume of calcite, (E) cell wall thickness intrafilament, (F) cell wall thickness interfilament. Previously published growth rates under ambient CO_2_ from both cultured (Blake and Maggs 2003) and natural samples (Freiwald and Henrich 1994; Halfar et al. 2000) shown by light pink box. The gray data points are from 3 months with 10 months shown as blue data points. All 3-month data as previously published by Ragazzola et al. 2012. Number of replicates *n* = 4; horizontal error bars display the standard deviation of μatm CO_2_ during the incubation period. Vertical error bars display the standard deviation of measurements within the same treatment.

For scanning electron microscopy (SEM), longitudinal sections of three different thalli for each treatment were cleaned with high pH distilled water and dried at 50°C for 24 h. The samples were then mounted on aluminum stubs and coated with a gold/palladium mixture (80%/20%). The samples were examined with a CamScan-CS-44. The extension rate was calculated by measuring from the growth tip to the stained section (i.e., the total growth after 10 months). In addition, the inter- and intrafilament cell wall thickness (Fig. 1A) was taken three cells from the growth edge to avoid newly deposited material which may not be fully calcified. These measurements were performed using ImageJ analysis software (Rasband 2008).

### TOMCAT (Tomographic Microscopy and Coherent rAdiology experimenTs)

Synchrotron-based X-ray Tomographic Microscopy was used as a nondestructive, high-resolution quantitative volumetric investigation of *L. glaciale* after 10 months. These measurements were performed at the TOMCAT beamline at the Swiss Light Source, Paul Scherrer Institut, Villigen, Switzerland (Stampanoni et al. 2006). For each tomographic scan, 1501 projections over 180 degrees were acquired at energy of 15 keV with an UPLAPO 20× objective (field view of 0.75 × 0.75 mm^2^; pixel size 0.37 × 0.37 μm^2^) and an exposure time of 300–400 msec. Projections were rearranged into corrected sinograms, and reconstructed using optimized FFT (Fast Fourier Transform) transformations and gridding procedures (Marone et al. 2010). The final data was exported as TIFF images (8bit 2 × 2 binned). Further processing to produce 3D isosurface and sample thickness was done using Avizo (Mercury Computer Systems Ltd., Chelmsford, MA). To determine the amount of calcite deposited in a set volume, three cubes from each treatment was selected with length of 80 μm giving a total volume 5.12 × 10^5^ μm^3^ (Fig. 1B). The cubes were taken three cells from the growth edge, as previously described.

### Electron microprobe

The algae were embedded in resin, polished, and carbon coated for element analysis. Elemental distribution was mapped with a JEOL JXA 8200 electron super probe using an accelerator voltage of 15.0 kV, a beam current of 50 nA, beam diameter 2 μm, dwell time 20 msec, and 10 accumulations. Three parallel transects, measuring Mg and Ca, were taken along the line of growth and averaged. Elemental concentrations were calibrated against known standards for Mg (glass VG-2-standard reference value: 4.05 wt%) and Ca (calcite-standard reference value: 40.11 wt%).

Mg/Ca was converted to temperature using the equation of Kamenos et al. 2008 (*T*_°C_ = 0.75 MgCO_3_ [mol%] −3.26) for *L. glaciale*.

### Data analyses

In order to compare the variability of the growth rate, inter- and intrafilament wall thickness within 10 month and the difference between 3 and 10 months, a two-way analyses of variance (ANOVA) was performed (Table 2). One-way ANOVA was used to test differences in the volume of calcite deposited during the culturing at different CO_2_ concentrations. To test if there was any difference on the Mg content before the experiments (so testing the natural variability prior the incubation period), Kruskal–Wallis was performed. Post hoc testing (Holm–Sidak test) was used to identify which treatments were significantly different from each other. To test the difference in Mg before (calcite deposited in natural environment) and after (calcite deposited during the experiment) the Alizarin staining, Mann–Whitney test was performed (Table 2).

**Table 2:** Statistic

	*P*	df	MS	*F*
Growth rates				
Two-way ANOVA				
CO_2_ treatments	ns	3	0.028	2.538
Months	***	1	0.628	56.576
CO_2_ treatments x months	***	3	0.083	7.471
Residual 56				
All pairwise comparison: Holm–Sidak Method				
*10 months*	566 μatm	770 μatm	1024 μatm	
408 μatm	**	***	***	
566 μatm	–	ns	ns	
770 μatm	–	–	ns	
Cell wall thickness (intra filaments)				
Two-way ANOVA				
CO_2_ treatments	ns	3	0.012	0.350
Months	***	1	1.907	54.883
CO_2_ treatments x months	***	3	0.297	8.550
Residual 24				
All pairwise comparison: Holm–Sidak Method				
*10 months*	566 μatm	770 μatm	1024 μatm	
408 μatm	ns	ns	ns	
566 μatm	–	ns	ns	
770 μatm	–	–	ns	
Cell wall thickness (inter filaments)				
Two-way ANOVA				
CO_2_ treatments	***	3	0.187	15.627
Months	***	1	1.672	139.746
CO_2_ treatments x months	***	3	13.482	13.482
Residual 24				
All pairwise comparison: Holm–Sidak method				
*10 months*	566 μatm	770 μatm	1024 μatm	
408 μatm	ns	ns	ns	
566 μatm	–	ns	ns	
770 μatm	–	–	ns	
Volume of Calcite				
One-way ANOVA				
*10 months*				
CO_2_ treatments	ns	3	3.97E+09	3.400
Mg/Ca (Before/After the Alizarin staining)				
Mann–Whitney				
*10 months*	P			
408 μatm	ns			
566 μatm	ns			
770 μatm	***			
1024 μatm	***			

ns, *P* > 0.05; *P < 0.05; **P < 0.01;***P < 0.001.

## Results

The growth rates of the specimens cultured under ambient CO_2_ (Fig 1C) is similar to that observed in the field, that is, under natural conditions (Halfar et al. 2000; Foster 2001; Blake and Maggs 2003; Adey et al. 2005; Kamenos et al. 2008). Growth of *L. glaciale* occurred under all the CO_2_ treatments including undersaturated conditions, albeit at lower rates. From the 10 months experiments, there was no significant difference between the growth rate of the three elevated CO_2_ treatments, with ∼60% decrease compared to the control (Table 2, Fig. 1C).

The volume normalized amount of calcite deposited shows no significant difference between the control and different CO_2_ treatments (Fig. 1D), as does the thickness of the interfilaments and the intrafilament cell walls (Fig. 1E, F) (Table 2).

We compared the results of the two phases of the experiment (3 and 10 months). The linear growth rates (i.e., extension rates) of the controls after 3 and 10 months were similar (1.02 mm/year and 0.93 mm/year, respectively) with no significant difference. The growth rate after 10 months compared to 3 months was significantly lower at all the elevated *p*CO_2_ treatments (Fig 1C). As the samples were stained only at the beginning of the experiment, and observations after 3 months showed that growth rate was maintained, the reduction in growth rate calculated over the whole 10 months, is likely to be an overestimation for the latter part of the experiment.

The intra- and interwall thickness in the controls are the same between 3 and 10 months, but the elevated treatments at 10 months shows much thicker intra- and interwalls and are now not significantly different to the control (Table 2).

**Figure 2:**
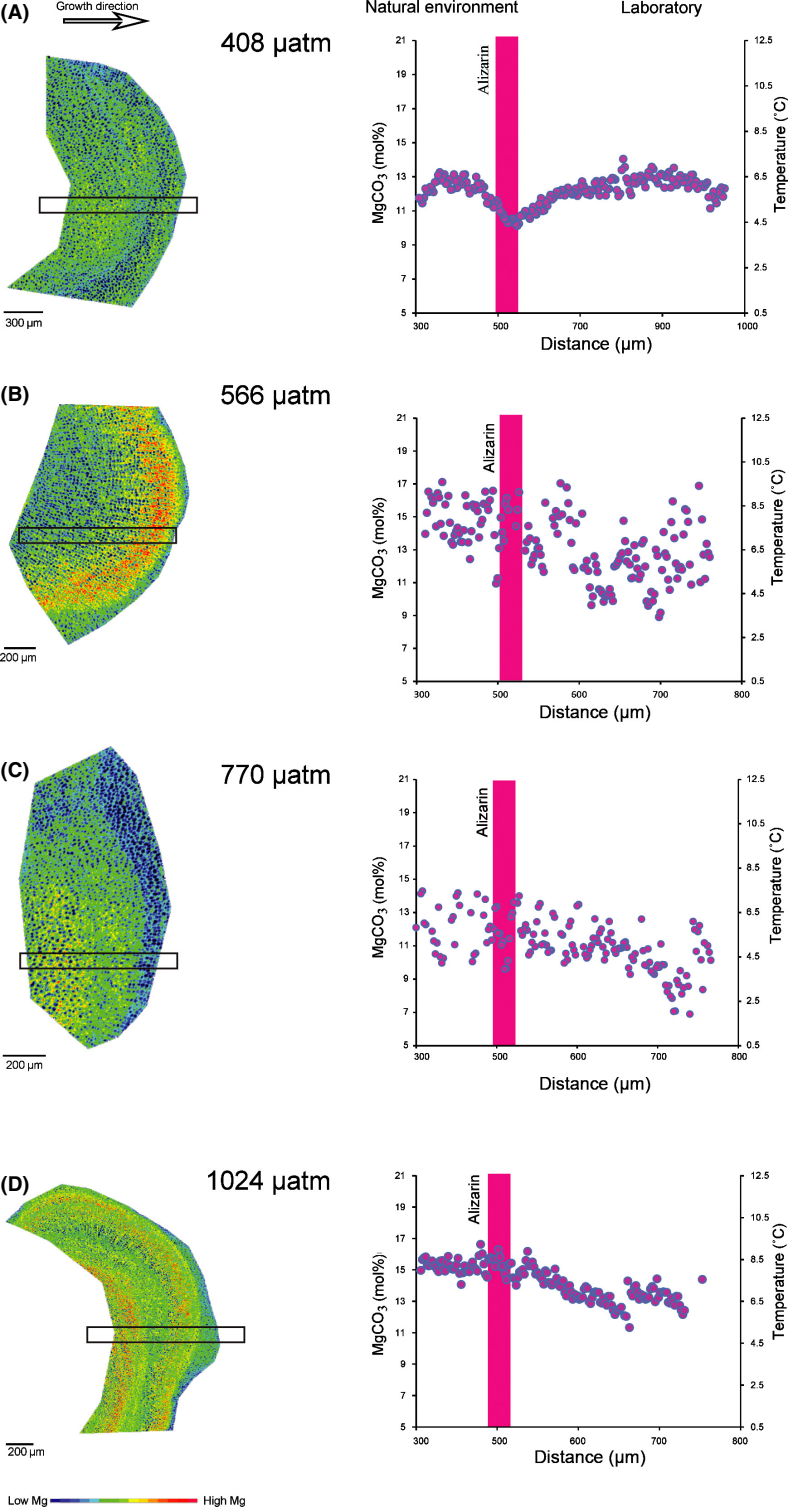
MgCO_3_ [mol%] under the four different *p*CO_2_ levels (*n* = 4). Three transects were taken from each electron probe map and averaged with data displayed to the right. The red line demarks the start of the experiment. Reconstructed temperature (°C) is shown on the secondary vertical axis using *Lithothamnion glaciale* temperature equation from Kamenos et al. 2008.

There is a significant difference in Mg concentration between all four specimens under natural conditions showing the natural variability (Fig. 2). There is no significant difference of Mg content before and after the Alizarin staining in the control, indicating that the laboratory control setup did not influence the Mg content. The control, grown at a temperature of 7 ± 0.5°C, gave a reconstructed temperature of 5.3 ± 0.6°C (SD) (Fig. 2). Only at the two higher CO_2_ treatments, 768 and 1000 μatm, a reduction in Mg content (11% and 15%, respectively) was observed (Table 2).

## Discussion

Comparison of short- and long-term experiments in other organisms such as cold water corals shows that the response of the organism changes with time (Form and Riebesell 2012). Thus suggesting that longer term experiments provide a better analog for understanding the organisms response to anthropogenic ocean acidification and provide a more accurate projection of future impact. In coralline algae, short-term experiments, on the order of days and weeks, have shown a decrease in growth rates (Kuffner et al. 2008; Büdenbender et al. 2011; Burdett et al. 2012) as a function of ocean acidification while longer term experiments over several months (including the 3 month experiment) have shown sustained growth rates (Martin and Gattuso 2009; Ragazzola et al. 2012; Martin et al. 2013). The comparison between the 3-month experiment (Ragazzola et al. 2012) and this study show that there are different phases during acclimatization with a different response in cell wall thickness and growth rate.

The cell wall thickness (both inter and intra filaments) at 10 months was equivalent to the control whereas at 3 months it was significantly thinner. However, growth rate at elevated CO_2_, which at 3 months was equivalent to the control, is now considerably reduced (Fig. 1). Our results from the same culturing experiment show different responses with time. A possible explanation is a shift from what could be termed a “passive” phase during the first 3 month to an “active” phase by the end of 10 months. During the “passive” phase, the increased energy requirement for calcification due to higher CO_2_ results in a reduction in the amount of calcite deposited in each cell wall. During the “active phase,” *L. glaciale* reduces its growth rate, but the cell wall structure is now maintained. The cell wall thickness varies seasonally with smaller but more calcified cells deposited in winter, and longer but less calcified cells in summer (Foster 2001). This different calcification pattern is induced by temperature and light (Martin et al. 2013; Kamenos and Law 2010). The algae were collected at the beginning of the summer and were kept in summer condition for 10 months in order to avoid a disruption of the circadian rhythms. The stable temperature and light during the experiments helped to isolate the effect of high CO_2_ in the thallus structures. The results of this study indicate that seawater chemistry can drive phenotypic plasticity in coralline algae. The ability to change the energy allocation between cell growth and structural support is a clear adaptive response of the organism.

There is no difference in cell wall thickness and growth rate between the different CO_2_ treatments: perhaps reflecting an adaptive response of the algae. It is also possible that adding other variables, such as temperature or different light period could increase the difference between the treatments due to synergistic effect (Martin et al. 2013).

Physiological responses to combat the effects of ocean acidification have previously been shown for other organisms such as *Amphiura filiforme* and other echinoderms (Shirayama and Thornton 2005; Wood et al. 2008). All the studies, including our, have shown that maintaining the skeletal integrity is one of the main priority of marine organisms living in high CO_2_ environment. For *A. filiformis* the trade-off between maintaining skeletal integrity and arm function will affect movement and as a consequence both feeding and respiration. In coralline algae there is a possible shift in the energy budget from growth extension to maintaining structural integrity, but the ecophysiological costs are still unclear. We are unable with this experiment to determine whether simply a reallocation of the energy budget from growth rate to wall thickness takes place, or if a more complex reallocation could occur within key processes such as reproduction or photosynthesis. It has been shown that photosynthesis can stimulate calcification and that its increase could offset the CaCO_3_ dissolution in different calcifying algae in response to increased CO_2_ (Borowitzka 1982; Gattuso et al. 1999; Hoffman et al. 2012; Johnson et al. 2012).

In addition to observing the morphology and growth rate of the coralline algae, we also examined the Mg content in the skeleton. This is particularly important for two reasons: (1) Mg incorporation is influenced by temperature and thus is applied as a temperature-proxy (2) Mg content has been shown to decrease in response to acidified conditions (Ries 2011) thereby reducing the solubility of the skeleton. The temperature reconstructed for the cultured control specimen was lower than the actual temperature (5.3 ± 0.6 vs. 7.7 ± 0.2°C [SD]). However, prior to the experiment (natural growth) we observe a high degree of heterogeneity in Mg content, which could account for the observed offset. To look at the impact of ocean acidification on Mg we compare the thallus before and after the Alizarin staining. In our experiment we found a decrease in Mg content only at the two highest CO_2_ treatments. As the growth rates are not significantly different in any of the treatments, it suggests that the growth rate and Mg content are not inherently linked, in agreement with Ries (2011).

The change in Mg alters the material properties of the calcite: increasing the solubility of the skeleton and altering the mechanical properties as a Mg-enriched matrix increases deformation resistance (i.e., has a greater resistance to applied stress) (Ma et al. 2008). This is particularly important, as coralline algae create important habitats by providing a hard substrate and thereby increasing the biodiversity of ecosystems (Foster 2001; Steller et al. 2003). The maintenance of structural integrity is important against predator and/or wave action. A reduction in the growth rate will change the mass balance, that is, the rate of calcification versus breakdown and dissolution of calcium carbonate. Thus, a reduction in growth rates under high CO_2_ could have implications at the ecosystem level.

Phenotypic plasticity may represent a strong selective advantage in such a biologically heterogeneous environment allowing algal species to persist in changing habitats while maintaining the potential to rapid morphological responses to unpredictable spatial or temporal fluctuations in climate. The response of coralline algae indicates a high degree of plasticity which is likely to increase its ability to survive in a high CO_2_ world.
